# Polymer Bionanocomposites Based on a P3BH/Polyurethane Matrix with Organomodified Montmorillonite—Mechanical and Thermal Properties, Biodegradability, and Cytotoxicity

**DOI:** 10.3390/polym16182681

**Published:** 2024-09-23

**Authors:** Beata Krzykowska, Łukasz Uram, Wiesław Frącz, Miroslava Kovářová, Vladimir Sedlařík, Dominika Hanusova, Maciej Kisiel, Joanna Paciorek-Sadowska, Marcin Borowicz, Iwona Zarzyka

**Affiliations:** 1 Department of Organic Chemistry, Faculty of Chemistry, Rzeszów University of Technology, Powstańców Warszawy 6, 35-959 Rzeszów, Poland; 2 Department of Inorganic and Analytical Chemistry, Faculty of Chemistry, Rzeszów University of Technology, Powstańców Warszawy 6, 35-959 Rzeszów, Poland; luram@prz.edu.pl; 3 Department of Material Forming and Processing, Faculty of Mechanical Engineering and Aeronautics, Rzeszów University of Technology, Powstańców Warszawy 8, 35-959 Rzeszów, Poland; wf@prz.edu.pl; 4 Centre of Polymer Systems, University Institute, Tomas Bata University in Zlin, Tr. T. Bati 5678, 76001 Zlin, Czech Republic; kovarova@utb.cz (M.K.); sedlarik@utb.cz (V.S.); d_hanusova@utb.cz (D.H.); 5 Department of Industrial and Materials Chemistry, Faculty of Chemistry, Rzeszów University of Technology, Powstańców Warszawy 6, 35-959 Rzeszów, Poland; m.kisiel@prz.edu.pl; 6 Department of Chemistry & Technology Polyurethanes, Faculty of Materials Engineering, Kazimierz Wielki University, JK Chodkiewicza Street 30, 85-064 Bydgoszcz, Poland; sadowska@ukw.edu.pl (J.P.-S.); m.borowicz@ukw.edu.pl (M.B.)

**Keywords:** polyester, polyurethane, structure–properties relationship, mechanical properties, thermal stability, biodegradability, cytotoxicity

## Abstract

In the present work, hybrid nanobiocomposites based on poly(3-hydroxybutyrate), P3HB, with the use of aromatic linear polyurethane as modifier and organic nanoclay, Cloisite 30B, as a nanofiller were produced. The aromatic linear polyurethane (PU) was synthesized in a reaction of diphenylmethane 4,4′-diisocyanate and polyethylene glycol with a molecular mass of 1000 g/mole. The obtained nanobiocomposites were characterized by the small-angle X-ray scattering technique, scanning electron microscopy, Fourier infrared spectroscopy, thermogravimetry, and differential scanning calorimetry, and moreover, their selected mechanical properties, biodegradability, and cytotoxicity were tested. The effect of the organomodified montmorillonite presence in the biocomposites on their properties was investigated and compared to those of the native P3HB and the P3HB-PU composition. The obtained hybrid nanobiocomposites have an exfoliated structure. The presence and content of Cloisite 30B influence the P3HB-PU composition’s properties, and 2 wt.% Cloisite 30B leads to the best improvement in the aforementioned properties. The obtained results indicate that the thermal stability and mechanical properties of P3HB were improved, particularly in terms of increasing the degradation temperature, reducing hardness, and increasing impact strength, which were also confirmed by the morphological analysis of these bionanocomposites. However, the presence of organomodified montmorillonite in the obtained polymer biocomposites decreased their biodegradability slightly. The produced hybrid polymer nanobiocomposites have tailored mechanical and thermal properties and processing conditions for their expected application in the production of biodegradable, short-lived products for agriculture. Moreover, in vitro studies on human skin fibroblasts and keratinocytes showed their satisfactory biocompatibility and low cytotoxicity, which make them safe when in contact with the human body, for instance, in biomedical applications.

## 1. Introduction

Polyurethanes are a class of polyaddition polymers with a wide range of industrial applications that includes linear, branched, and cross-linked products [1]. They consist of three monomers: a diisocyanate, a polyol, and a chain extender [2]. The main criterion that distinguishes polyurethanes from other plastics is their density [3]. Polyurethanes can be available as both relatively rigid plastomers and flexible elastomers with a solid or foamed structure [3].

Polyurethanes have excellent mechanical strength; toughness; good resistance to abrasion, corrosion, and chemicals; and flexibility at low temperatures [4]. One of the most important categories of polyurethanes is polyurethane elastomers, which have been widely incorporated into various engineering products and have been proven to offer very good properties [5]. They are malleable polymers and can be easily processed, either by extrusion or injection molding, and offer high recyclability [6]. As a general rule of thumb, for linear polymers, all properties, such as tensile strength, elongation, elasticity, melting point, glass transition temperature, and molecular weight, increase up to a limited value, after which all properties remain virtually unchanged. This behavior is useful for linear polyurethanes—polyurethane elastomers [7].

Today, polyurethanes are one of the most common, versatile, and researched materials in the world [6,8]. These materials combine the durability and strength of metals with the flexibility of rubber, making them suitable for replacing metals, plastics, and rubber in some engineering products [8,9]. They are widely used in biomedicine, construction, automotive, textile, and many other industries due to their excellent properties in terms of hardness, elongation, and strength [10,11,12].

Polyurethanes are widely used in biomedical applications due to their good physical and mechanical properties, as well as their relatively good biocompatibility and anti-coagulation properties [13]. Initially, polyurethanes in medical applications were used as artificial skin, vascular grafts, nerve connections, bone grafts, and articular cartilage repair materials [14]. Today, technologies such as computer-aided design (CAD)/computer-aided manufacturing (CAM) [2] and 3D printing [2,14] are being used to produce polyurethanes for medical purposes. Polyurethane plastics have found medical applications as blood vessel implants [15] and temporary membranes that help restore the function of body organs that have been damaged in accidents [16].

Polyurethanes are used in the field of gynecology, reconstructive surgery materials, and cardiovascular devices. In addition, polyurethanes are used in the fields of wound dressings, tissue engineering, and controlled drug release [2]. Their advantage over other materials lies in their good mechanical properties and biostability [17,18]. Biodegradable polyurethanes based on castor oil [19], carbohydrates [20], chitosan (with antimicrobial properties) [21], and electroactive polyurethanes in cardiac tissue engineering [22] are also used.

However, the most common application is in short-term implants. Incorporating polyurethanes into medical-related applications is economically viable and at the same time introduces robust and long-lasting materials [23]. Due to their excellent mechanical properties and biocompatibility, linear polyurethanes can be used to modify the properties of polyhydroxyalkanoates.

In this work, polyurethane produced with the use of 4,4′-diisocyanate diphenylmethane (MDI) and polyethylene glycol was applied to modify the properties of poly(3-hydroxybutyrate), P3HB. To further modify the properties of P3HB, a layered organo-modified aluminosilicate, Cloisite 30B, was used as a nano-additive. The effect of the used modifiers on the mechanical and thermal properties, biodegradability, biocompatibility, and cytotoxicity of P3HB was investigated.

## 2. Materials and Methods

### 2.1. Materials

P3HB was supplied by Biomer (Krailling, Germany). Its molecular weight was in the range of 400–1200 kDa and dispersion of *Mw·Mn*^−1^ = 5.72, as determined by exclusion chromatography in chloroform. Its melt flow rate was 0.11 g (10 min)^−1^ (180 °C at 2.16 kg). Cloisite 30B, which is a montmorillonite modified with methylbis(2-hydroxyethyl)tallowammonium cations, was provided by Southern Clay Products Inc. (Gonzales, TX, USA).

Polyurethane aromatic rings based on diphenylmethane 4,4′-diisocyanate (PU-MDI) were prepared according to a previously described procedure [24]. The reaction was carried out in anhydrous acetone at 60 °C under a nitrogen atmosphere. MDI dissolved in acetone was dropped into a solution of polyethylene glycol with a molecular weight of 1000 g with dibutyltin dilaurate (DBTL) in acetone. The progress of the reaction was checked by the content of the NCO groups. The final product was purified with cold acetone by precipitation.

Normal human fibroblasts (BJ cell line), a penicillin and streptomycin solution, and Eagle’s Minimum Essential Medium (EMEM) were purchased from American Type Culture Collection (ATCC, Manassas, VA, USA). Human immortalized keratinocytes (HaCaTs) were obtained from Cell Lines Service (Eppelheim, Germany). Dulbecco’s Modified Eagle’s Medium (DMEM) and fetal bovine serum (FBS) were purchased from Corning (New York NY, USA). Trypsin–EDTA solution was delivered by Gibco Thermofischer Scientific (Waltham, MA, USA). Phosphate-buffered saline (PBS) with or without magnesium and calcium ions, crystal violet, 0.4% trypan blue solution, and sterile syringe filters of 0.22 µm were provided by Sigma–Aldrich (St Louis, MO, USA). Cell culture flasks were from Corning Incorporated (Corning, NY, USA) and 6-well plates from Nunc (Roskilde, Denmark).

### 2.2. Nanobiocomposite Preparation

**Figure 1:**
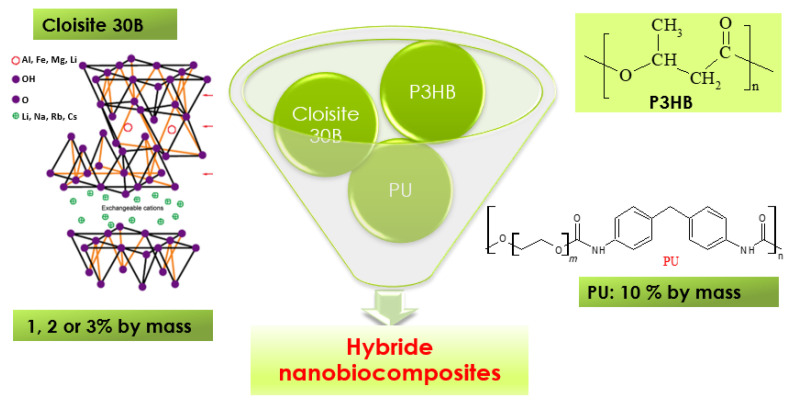
Scheme of nanocomposite production.

Nanobiocomposites were prepared by combining P3HB and 10 wt.% of polyurethane (PU) with 1, 2, or 3 wt.% Cloisite 30B clay (designated as C10-1, C10-2, and C10-3, respectively), as described in Table 1, in a co-rotating twin-screw extruder (Figure 1). Initially, P3HB was dried at 40 °C under reduced pressure for 30 min. The nanoclay was then dispersed in water using an ultrasonicator at room temperature for 2 h. In the next step, the nanoclay was filtered and dried at 40 °C under reduced pressure. Native P3HB and P3HB with the addition of 10 wt.% PU (designated C10) were also extruded to obtain reference materials. The twin-screw extruder, equipped with four temperature-controlled zones, was programmed from 150 to 185 °C. The screw diameter was 12.5 mm and the ratio L·D^−1^ was 24. The screw speed during the extrusion process was 40 rpm. The extrusion conditions were determined experimentally.

### 2.3. Analytical Methods

#### 2.3.1. Scanning Electron Microscopy

To characterize the phase morphology of all nanobiocomposites and native P3HB, and the composition of P3HB-PU, scanning electron microscopy (SEM) was used. The investigated surface was coated with a thin layer of gold by a sputtering process. A HITACHI S-3400 N scanning electron microscope (Tokyo, Japan) with different image magnifications and an accelerated voltage of 10 kV was used.

#### 2.3.2. SAXS Analysis

In order to verify the nanostructure of the biocomposites, small-angle X-ray scattering (SAXS) measurements were performed. The tests were carried out at room temperature using a Bruker SAXS Nanostar-U X-ray diffractometer. The comparative spectra of press-formed samples were studied in the transmission mode (coupled h·2h^−1^) to check the effect of the nanoparticle orientation. The slit system allowed for the collection of a deflected beam with a divergence angle of less than 0.05. The measurements were carried out in the range from 0 to 28° at 2Θ. The small-angle goniometer was coupled to a Cu-filtered radiation source Kα (1.54 Å) in a closed tube, operating at 50 kV and 0.6 mA.

#### 2.3.3. Fourier Transformation Infrared Spectroscopy

The infrared spectra of the native P3HB, PU, polymer composition P3HB-PU, and hybrid nanobiocomposites were measured with the use of an ALPHA FT-IR spectrometer. The measurements were made in the wave number of 400–4000 cm^−1^. The spectra were recorded at a resolution of 2 cm^−1^ using the ATR technique.

#### 2.3.4. Thermogravimetric Analysis

The thermogravimetric analysis (TG) of P3HB and its nanocomposites were carried out using a Mettler Toledo TGA/DSC1 apparatus. The measurements were carried out using the following conditions: sample mass of ~10 mg, gas flow of 50 mL·min^−1^, 150 µL open alumina pan, and in the temperature range of 25–700 °C at a heating rate of 10 °C·min^−1^ in a nitrogen atmosphere.

#### 2.3.5. Differential Scanning Calorimetry (DSC) Analysis

The temperature dependence of the heat flow rate was obtained using a differential scanning calorimeter from Mettler Toledo. In order to obtain highly accurate measurements, the temperature calibration and calibration of the heat flow rate were performed with respect to the melting parameters of indium, i.e., the initial temperature was 429.6 K and the heat of fusion ΔHf = 28.45 J·g^−1^ (3.28 kJ·mol^−1^). All analyses were carried out in a nitrogen atmosphere at a constant flow rate of approximately 50 mL·min^−1^. In order to improve the quality of the results obtained, a calibration of the heat capacity was performed. Three measurements were carried out for each sample, including a blank sample (reference) and composites of real samples, calibrated with a sapphire. The accuracy of the heat capacity measurements was approximately 3%. The experiments were carried out in the temperature range from −90 °C to 230 °C at a heating rate of 10 °C·min^−1^. The data were collected from the second heating scan.

#### 2.3.6. Strength Tests

Specimens for the strength tests were obtained by injection molding on a DrBoy 50 injection molding machine equipped with the Priamus production monitoring and control system. The specimens were made in accordance with PN EN ISO 527 (1998) [25]. The samples were molded according to the same injection parameters, which made it possible to assess changes in the plastic state parameters in the mold.

The tensile tests were carried out at room temperature on a Zwick Roell Z030 machine equipped with an automatic multiextensometer, at a machine gripping speed of 50 mm·min^−1^. The Young’s modulus was determined at a speed of 1 mm·min^−1^. Tests were carried out on at least seven specimens in a series. Only those specimens showing the best parameters were selected for testing.

The hardness of the nanobiocomposite samples obtained was determined using the Brinell method in accordance with PN-EN ISO 6506-1:2006 [26]. The samples were made in accordance with PN-EN ISO 527-1 [27].

The tensile strength of the specimens was tested with an INSTRON impact hammer in accordance with ISO 8256-A-4J [28]. The notch was cut on both sides according to method A of the aforementioned standard.

#### 2.3.7. Biodegradability Testing

The biodegradability of P3HB-based composites was tested using the OxiTop^®^ Control S6 device from WTW-Xylem (San Diego, CA, USA), which used a respirometric method of measuring the oxygen demand necessary for the aerobic biodegradation of polymeric materials in the soil. The measurement of oxygen consumed was expressed using the biological oxygen demand (BOD) value, which is expressed as the number of milligrams of captured oxygen per unit mass of the tested polyurethane material. The OxiTop^®^ Control S6 apparatus consisted of glass bottles with a capacity of 510 mL, equipped with rubber covers and OxiTopC measuring heads, with which BOD was measured. They allowed for pressure measurements in the range from 500 to 1350 hPa with an accuracy of 1% at temperatures from 5 °C to 50 °C. The set also included the OC 110 controller, which was used for communication between the measuring heads, the user, and the Achat OC computer 10.1 software (WTW-Xylem, San Diego, CA, USA), used to interpret the obtained measurement results.

The biodegradability test of P3HB and its composition with linear polyurethane containing aromatic rings and their composites with Cloisite 30B was performed according to the ISO 17556:2012 standard [29]. Sieved and dried garden soil with a high humus content and physicochemical parameters, such as humidity 5% (according to ISO 11274 [30]), pH 6 (according to ISO 10390 [31]), and grain diameter <2 mm, was used as the biodegradation environment. Then, it was weighed to the amount of 200 g and put into the bottle from the OxiTop set. In the next step, 100 mL of distilled water was added. The mixture was mixed, and then, approximately 200 mg of solid test sample was added and mixed again. The exact mass of the tested sample was read and saved. After preparing the system, a rubber quiver was placed in which 2 solid NaOH pellets were placed and the measuring head was screwed on. This step was also repeated for the remaining OxiTop bottles. The research set was placed in an incubator maintaining a constant temperature of 20 ± 0.2 °C and initially thermostated at this temperature for 2 h. Then, the BOD measurement program was started using the OC 110 controller and left in the incubator at a constant temperature of 20 ± 0.2 °C for 28 days. The measurement results were read from the measuring heads every 2–3 days.

The set of test samples included two reference systems (positive and negative), one blank sample, and samples of the tested materials. A mixture of soil and water without biodegradable material was used as a blank test. The positive test was an easily biodegradable natural polymer, starch, while the negative test was a completely non-biodegradable polymer, polyethylene. Five types of P3HB-based samples were used for the study: native P3HB, P3HB-PU composition, and 3 P3HB composites containing 10 wt%. PU and increasing contents of Cloisite 30B in the amount from 1 to 3% by weight. In accordance with the ISO 17556:2012 standard, all samples tested were of comparable size because the absolute degree of degradation depends on the form and shape of the material.

The biochemical oxygen demand (BOD) for a single OxiTop^®^ Control S6 bottle was determined using the formula considering the BOD of the tested system, corrected for the BOD of the soil itself, and considering the concentration of the tested material in the soil.

$${BOD}_{S}=\frac{{BOD}_{x}\;-{\;BOD}_{g}}{c}$$

where S—number of days of measurement; BOD_S_—biochemical oxygen demand of the analyzed material during S days [mg·L^−1^]; BOD_x_—measurement result for system x [mg·L^−1^]; BOD_g_—measurement results for soil only (without sample) [mg·L^−1^]; and c—sample concentration in the tested system [mg·L^−1^].

The degree of biodegradation of the polymer material was determined based on the equation:

$$D_{t}=\frac{{BOD}_{S}}{TZT}\cdot 100\%$$

where D_t_—degree of material biodegradation [%]; TOD—theoretical oxygen demand [mg·L^−1^].

The theoretical oxygen demand for each system was calculated using the formula included in the standard. It was assumed that, as a result of the biodegradation process in aerobic conditions, the carbon contained in the tested compound turns into CO_2_, hydrogen into H_2_O, phosphorus into P_2_O_5_, sulfur into SO_3_, and nitrogen in NH_3_, and chlorine in HCl. For a compound with a known formula containing the elements C, H, Cl, N, S, P, Na, and O, the TOD value can be calculated according to the equation:

$$TOD=\frac{16[2c+0.5\left(h-cl-3n\right)+3s+2.5p+0.5k-o]}{M_{r}}$$

where c, h, p, s, n, cl, k, o—number of individual elements in a molecule of biodegraded material [-]; M_r_—mass of biodegraded material [g].

#### 2.3.8. Elemental Analysis

Elemental analyses (C, H, N) of the native P3HB, PU, P3HB-PU polymer composition, and hybrid nanobiocomposites were carried out on the analyzer Vario EL III C, H, N from Elementar.

#### 2.3.9. Cell Culture

The BJ normal human skin fibroblasts (ATCC, Manassas, VA, USA) were cultured in EMEM and HaCaT human immortalized keratinocytes (CLS, Neuenstein, Germany) in a DMEM medium. Both media were supplemented with 10% heat-inactivated FBS, 100 U/mL penicillin, and 100 µg/mL streptomycin. The cells were cultured at 37 °C in an atmosphere of 5% CO_2_ and 95% humidity with the growth medium changed every 2–3 days and passaged at about 80% confluence with 0.25% trypsin-0.03% ethylenediamine-tetraacetic acid in calcium- and magnesium-free PBS. Cell morphology was monitored under a Nikon TE2000S inverted microscope with phase contrast (Tokyo, Japan). The number and viability of cells were estimated by the trypan blue exclusion assay with Automatic Cell Counter TC20™ (Bio-Rad Laboratories, Hercules, CA, USA).

#### 2.3.10. In Vitro Cytotoxicity Tests

The cytotoxicity of polymers was estimated with a direct contact toxicity assay, as described earlier [32]. Normal human fibroblasts (BJs) and immortalized keratinocytes (HaCaTs) were seeded in 6-well plates in the amount 3 × 10^5^ and 8 × 10^5^ cells/well, respectively, and incubated 24 h in 37 °C, 5% CO_2_, and 95% humidity. Then, 1 mL of fresh media with 10% FBS per well was added and thermally sterilized samples (10 × 10 mm × 5 mm thickness) were placed on a cell monolayer and incubated for 24 h. Then, samples were removed and cells were washed once with PBS and stained with 0.2% crystal violet in 2% ethanol for 30 min. After washing with distilled water (thrice), images of plates with stained cells were collected, and the reactivity zones were assessed with the ImageJ 1.49 software (NIH, Bethesda, MD, USA). Obtained results were interpreted by the grade of the reactivity zone described by U.S. Pharmacopeial Convention [33] and ISO 10993-12 [34]. Then, the solubilization of the stained cells was performed with 10% acetic acid in a shaker (1 mL/well, 10 min, 400 rpm, room temperature). The absorbance of the CV solutions from the individual samples were measured at 595 nm against 450 nm and against a blank sample (10% acetic acid) with a microplate reader (µQuantTM, BioTekInstruments, Inc. Winooski, VT, USA). The results are presented as a % of the control. All experiments were performed in triplicate.

#### 2.3.11. Statistical Analysis

Differences between the treated and non-treated control samples were estimated with the non-parametric Kruskal–Wallis test due to the lack of a normal distribution of data in the studied groups (analyzed with Shapiro–Wilk test). The Mann–Whitney U test was used to evaluate the differences between two studied cell lines incubated with the same samples. *p* ≤ 0.05 was regarded as statistically significant. All analyses, calculations, and figures were performed with the Statistica 13.3 software (StatSoft, Cracow, Poland).

## 3. Results and Discussion

Considering the obtained P3HB–PU polymer compositions described in our previous manuscript [24], and taking into account a further improvement in the physical properties of P3HB, hybrid nanobiocomposites were prepared with the use of P3HB as the matrix, with the addition of linear PU with aromatic rings as the modifier and an organomodified montmorillonite, Cloisite^®^30B, as a nanofiller. Based on the previous results, the polymer blends with the best thermal and mechanical properties were selected to further tests. It was selected the polymer blend based on PU obtained from the reaction of 4,4′-diphenylmethane diisocyanate and polyethylene glycol with the molecular weight of 1000 g. PU was used at 10 m/m%. In turn, Cloisite^®^30B was used at 1, 2, and 3 m/m%—at typical amounts to produce nanocomposites.

### 3.1. Results of SAXS Measurements

**Figure 2:**
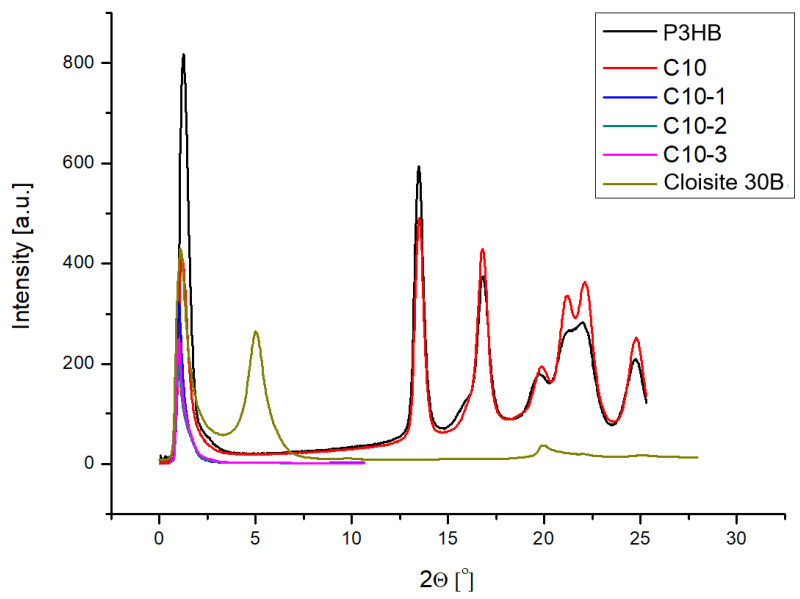
SAXS plots of nanocomposites containing 1, 2, and 3 wt.% of Cloisite 30B and 10 wt.% of PU (designated as C10-1, C10-2, and C10-3, respectively). The figure shows the reference diffractograms of the unfilled P3HB and the polymer composition of P3HB and 10 wt.% PU and pure Cloisite 30B.

In order to characterize the nanoclay structure in the prepared composites, the SAXS technique was used. Measurements of the obtained hybrid composites were carried out in the 2θ angle range from 0° to 28°, and the results are shown in Figure 2. The structure of montmorillonite (intercalated or exfoliated) was determined using the Bragg equation, which allows to calculate the values of the distance d between the nanoclay plates. Figure 2 illustrates the pattern of the Cloisite 30B nanofiller, showing a peak with a maximum of approximately 2θ = 4.92°, which means the distance between the nanoclay plates is d_001_ = 1.8 nm [35]. As can be seen, all the obtained composites do not have peaks in the range from 1 to 5° (Figure 2), which confirms the complete randomization and exfoliation of the nanofiller [36].

### 3.2. Results of FTIR Analysis

**Figure 3:**
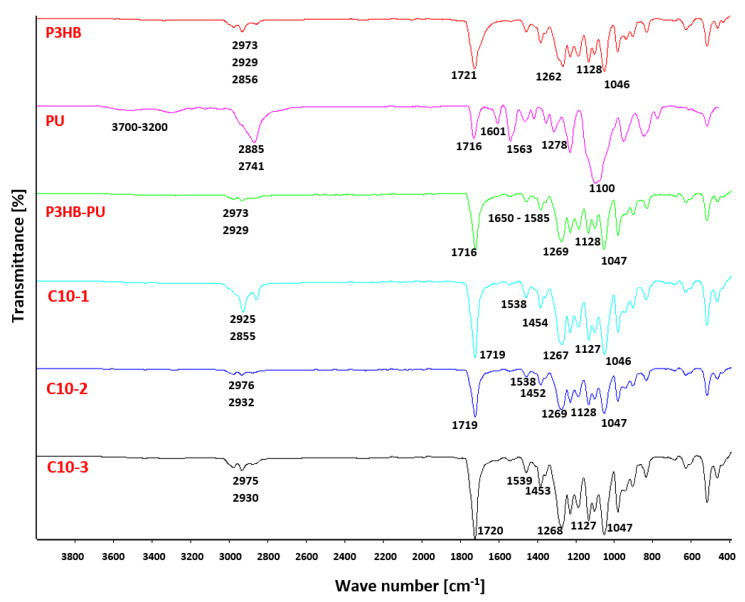
Set of FTIR spectra of a native P3HB, PU, and P3HB-PU polymer biocomposition containing 10 wt.% PU and bionanocomposites based on the P3HB-PU polymer matrix with 1, 2, and 3 wt.% of Cloisite 30B (C10-1, C10-2, and C10-3, respectively).

The compatibility of the components the tested materials P3HB, PU, and Cloisite 30B was checked by FTIR spectra measurements [37] and is shown in Figure 3. The FTIR spectrum of a native P3HB shows bands characteristic of esters, i.e., the band originating from the stretching vibrations of carbonyl groups at 1721 cm^−1^. There are also visible bands of asymmetric and symmetric vibrations of the C-O ester bonds at 1262 and 1128 cm^−1^, respectively. In addition, there is a band of C-OH stretching vibrations of secondary alcohols at 1046 cm^−1^, because the polyester chains are terminated with hydroxyl groups. The share of hydroxyl groups is small; therefore, the valence vibration band of O-H bonds is not observed above 3000 cm^−1^. In turn, at the wave numbers of 2929 and 2974 cm^−1^, two bands can be observed originating from the stretching vibrations of asymmetric and symmetric C-H bonds of the methyl and methylene groups of P3HB, respectively.

The FTIR spectrum (Figure 3) of aromatic linear polyurethane (PU) shows a broad band in the range of 3700–3200 cm^−1^, originating from the stretching vibrations of the N-H bonds of urethane groups. At 2885 and 2741 cm^−1^, the asymmetric and symmetric vibration bands of the C-H bonds of the methylene groups of the aliphatic fragment of the PU chain are observed. The stretching vibration band of the C=O groups of urethanes occurs at 1716 cm^−1^, and at a wave number of 1601 cm^−1^, the bending vibration band of the N-H bonds of polyurethane. However, at 1278 and 1100 cm^−1^, asymmetric and symmetric vibration bands of C-O bonds of urethane groups appear, respectively. The bands in the range of 1597–1413 cm^−1^ refer to the skeletal vibrations of aromatic rings.

**Figure 4:**
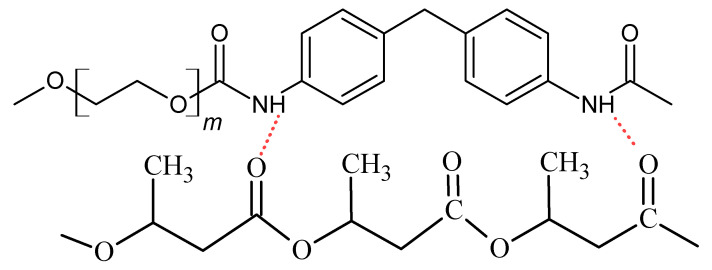
Scheme of hydrogen bond formation between P3HB and PU chains.

In the FTIR spectrum of the P3HB-PU polymer composition (Figure 3), there is a change in the shape of the bands compared to the spectrum of polyurethane above 3000 cm^−1^. There is very broad and a small intensity band. In the range of 3000–2800 cm^−1^, there are bands originating from asymmetric and symmetric vibrations of C-H bonds of the methyl and methylene groups of P3HB and PU, respectively. In the spectrum of the obtained composition, a characteristic band at 1716 cm^−1^ can also be noticed. This is a common band originating from the stretching vibrations of carbonyl groups occurring in ester and urethane groups (Figure 4). In the range of 1585–1680 cm^−1^, a multiplate band appears due to the bending vibrations of the amide N-H bonds due to the formation of intermolecular hydrogen bonds [38,39].

Similar to the spectra of P3HB and PU, in the range of 1040–1300 cm^−1^, there appear bands originating from the asymmetric and symmetric vibrations of the C-O bonds of the ester and urethane, respectively.

The FTIR spectra of the hybrid nanobiocomposites obtained by introducing organically modified montmorillonite (Cloisite 30B) (Figure 3) in the amounts of 1, 2, and 3% by mass were also analyzed. Some similarities and differences with respect to the individual polymers (P3HB and PU) or the polymer composition were observed regarding the intensity and position of the bands. In the range of 3600–3100 cm^−1^, broad, blurry bands of a low intensity can be observed, which slightly increase with the increase in the amount of Cloisite 30B in the nanobiocomposite. These bands are characteristic of the N-H groups of polyurethane and the OH groups of alcohols, which form hydrogen bonds between the urethane and hydroxyl groups of the modifier and the ester and hydroxyl groups of P3HB. The multiplicity of the band in the range of 1585–1680 cm^−1^ proves the formation of intermolecular hydrogen bonds [40]. Moreover, in all spectra of nanobiocomposites, there is a band with a wave number of 1719 cm^−1^, common to the stretching vibrations of the C=O bonds of the P3HB ester group and the PU urethane group. At 1538 cm^−1^, there is a band of the bending vibrations of the N-H bonds of urethane. Additionally, at the wave numbers of 1268 cm^−1^ and 1127 cm^−1^, there are typical bands of asymmetric and symmetric C-O vibrations of esters and urethanes.

The FTIR spectral analysis of the components prepared using polymer composites confirmed the interactions of PU with the Cloisite30B and P3HB matrix in the nanobiocomposites based on, e.g., hydrogen bonds.

### 3.3. Results of SEM Analysis

**Figure 5:**
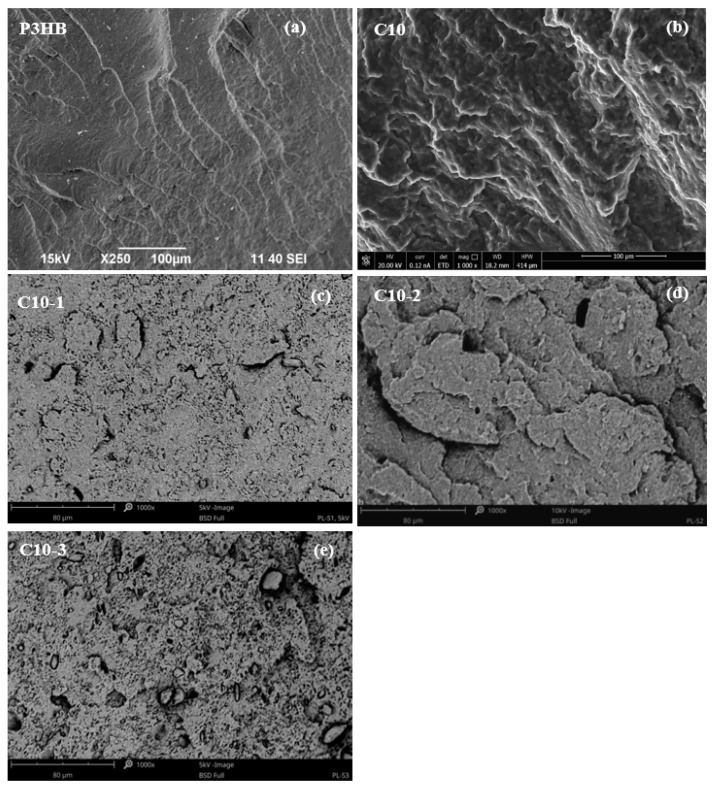
SEM micrographs of P3HB (**a**) and its polymer biocomposition containing 10 wt.% polyurethane (P3HB-PU, C10) (**b**), and nanobiocomposites with 10 wt.% PU and 1 wt.% (**c**), 2 wt.% (**d**), and 3 wt. % of Cloisite 30B (**e**) (C10-1, C10-2, and C10-3, respectively).

Figure 5 shows the SEM micrographs of the fracture surface of samples: polyester—P3HB (Figure 5a), P3HB-PU binary composition (Figure 5b) containing a 10 wt.% addition of polyurethane based on polyethylene glycol with a molar mass of 1000 g/mol, and hybrid composites based on P3HB-PU with different amounts of nanoclay Cloisite 30B (1, 2, and 3 wt.%), (Figure 5c,d). The images of the fracture surface of the destroyed samples make it possible to explain the mechanism of influence of the used modifiers on the mechanical properties of the tested biocomposites. The presented micrographs were obtained by scanning the fracture surface of the samples at the point of fracture as a result of the used force. The fracture surface of the polyester matrix shown in Figure 5a is slightly wavy and glassy, which suggests a regular and flexible crack propagation path, and the brittle fracture areas are arranged unidirectionally. Figure 5b shows the fracture of the binary composite made of P3HB modified with a 10 wt.% amount of the polyurethane modifier. It can be seen that the introduction of PU into the polyester matrix disrupts the continuity of its structure and results in the appearance of rough, wavy areas arranged in different directions. Their presence may suggest the interaction of the P3HB matrix with PU, resulting in the separation of the interacting biopolymer chains and the formation of the aforementioned rough areas. This shift may be related to the improvement in the mechanical properties of the binary composition compared to the matrix itself. For the two-component P3HB-PU composition, an increase in elongation and impact strength as well as a decrease in hardness and a slight decrease in strength were noted, which was related to the elasticizing effect of the polyurethane modifier [41]. The micrographs in Figure 5c–e document the structure of the hybrid biocomposites modified with 10 wt.% PU and various amounts of nanoclay Cloisite 30B. All hybrid biocomposites have a similar structure. Regardless of the modified aluminosilicate content, the integral components of the hybrids cannot be distinguished. The three-component composites have numerous micro- and macro-cracks and pores. The structure of these materials is phase-heterogeneous. The introduction of nanoclay into the composition increases the hardness of the materials produced and does not depend on the amount of montmorillonite introduced into the composites. The clay used only stiffens the manufactured materials, i.e., P3HB or P3HB-PU, resulting in their greater hardness.

The aforementioned phenomenon of the heterogeneity of the hybrid structure is highlighted mainly in composites containing clays at 1% or 3% by mass. The heterogeneity of the structure of the three-component materials indicates an insufficient compatibility of the components included in them and, in relation to the matrix and binary composition, results in the deterioration of some properties of the hybrid composites. It should be noted that the polyester matrix modified by 10 wt.% of PU and average, i.e., 2% by mass amount of clay, has a slightly different structure and is characterized by the smallest number of cracks and pores mentioned above than other hybrid samples. In the micrograph (Figure 5d), rough domains are forming, arranged in different directions. This therefore explains the better mechanical properties of the composite containing 2 wt.% modified with montmorillonite than those of the other hybrids (with 1% or 3% clay by mass). Therefore, it appears that the P3HB modification with 10% of PU and 2% by mass Cloisite 30B results in the most favorable mechanical properties compared to the properties of the other manufactured materials.

### 3.4. Mechanical Properties the Obtained Hybrid Nanobiocomposites

**Figure 6:**
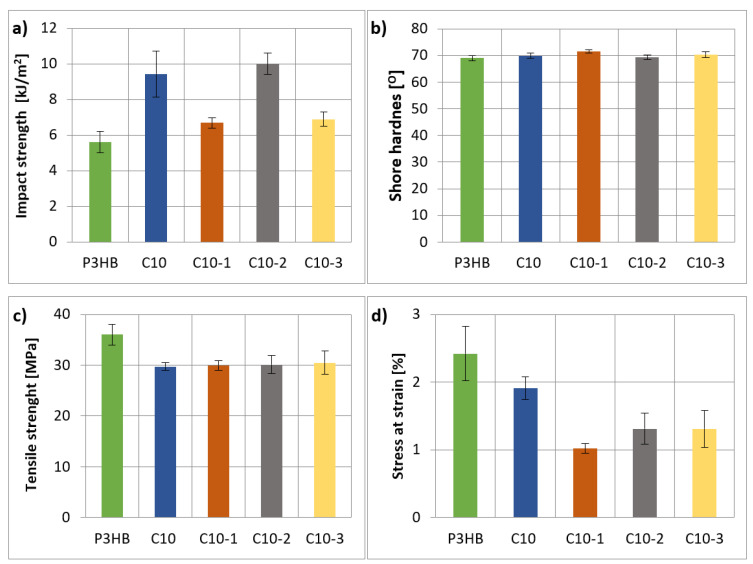
Graphs of the (**a**) impact strength, (**b**) hardness, (**c**) tensile strength, and (**d**) relative elongation at break of the extruded products—P3HB, P3HB-PU (C10) polymer composition, and polymer nanobiocomposites with 1, 2, and 3 wt.% Cloisite 30B (C10-1, C10-2, and C10-3, respectively).

Based on the obtained mechanical test results, plots were prepared showing the influence of the organic nanoclay content on the Charpy impact strength (Figure 6a), Shore hardness (Figure 6b), tensile strength (Figure 6c), and relative elongation at break (Figure 5d) of the prepared polymer nanobiocomposites compared to the P3HB-PU (C10) polymer composition and the native P3HB.

The impact strength value of all obtained polymer nanobiocomposites (Figure 6a) is much higher than that of the native P3HB, but not always higher than that of the P3HB-PU composition [42]. The nanobiocomposite containing 2 wt.% has the highest impact strength, higher than C10 about 6%. Compared to the native P3HB, the increase in the impact strength was approximately 80%.

The hardness of the produced polymer nanobiocomposites (Figure 6b) depends on the amount of organic nanoclay introduced. The nanobiocomposite containing 1% by mass Cloisite 30B had the highest hardness (71.5°), and it is higher than the hardness of the P3HB-PU (C10) composition and the native P3HB. Adding 2% by mass Cloisite 30B makes the nanobiocomposite (C10-1) have the lowest hardness, equal to the hardness of the native P3HB. The introduction of 3% by mass, Cloisite 30B (C10-3), causes a slight increase in the hardness to the level of the P3HB-PU polymer composition (C10). The presence of the polyurethane modifier and the nano-additive does not make the rigid structure of P3HB more flexible; therefore, the addition of the aromatic linear polyurethane and the nano-additive did not reduce the hardness of the obtained polymer nanobiocomposites.

Based on the graph presented in Figure 6c, it can be concluded that all produced nanobiocomposites based on P3HB with linear aromatic polyurethane are characterized by lower tensile strength values compared to the native P3HB. Both the P3HB-PU (C10) polymer composition and the nanobiocomposites have a tensile strength of approximately 30 MPa, which is a 1/6 decrease compared to 36 MPa for native P3HB. Paşcu at al. obtained similar results [43]. The value of tensile strength is still very good and acceptable for the intended application.

In the case of the relative elongation at break (Figure 6d), it can be seen that P3HB modified with linear aromatic polyurethane (C10) has a reduced value of this parameter. The introduction of Cloisite30B causes the relative elongation at break to decrease even more, especially for C10-1. An increase in the nano-additive content causes an increase in the relative elongation at break to a constant level of 1.3%, independent of the amount of additive.

### 3.5. Thermal Properties of the Obtained Nanobiocomposites

The thermal stabilities of the produced hybrid polymer nanobiocomposites, the native P3HB, and of that composed with PU were determined using thermogravimetric analysis. The results of the analysis are presented in Table 2. The temperature of the onset decomposition of all nanobiocomposites, as well as the temperature of 5 and 10% weight loss, are much higher than of the native P3HB and comparable to that of the P3HB-PU composition.

The introduction of clays and increasing their amount do not significantly affect the thermal stability of the obtained nanobiocomposites, and the values of the onset decomposition temperatures for all tested samples are similar. The temperature of the maximum decomposition rate of P3HB is close to the temperature of the maximum decomposition rate of the prepared compositions and is in the range of 293–295 °C. The residual mass of the analyzed polymer mixtures at a temperature of 600 °C increases with the increase in the amount of Cloisite 30B added and does not exceed 3% by mass. The preparation of P3HB-based hybrid polymer nanobiocomposites with aromatic linear polyurethane and organomodified montmorillonite increases slightly the thermal stability of the obtained materials compared to that of the P3HB-PU composition.

Thermal stability is an important parameter to consider in order to determine the processing temperature (extrusion, injection) of synthetic and biodegradable thermoplastic polymer matrices [44]. In this context, our hybrid nanobiocomposites containing polyurethane chains and organically modified montmorillonite can be processed at higher temperatures, up to a maximum of about 252 °C.

### 3.6. Thermal Parameters from DSC Measurements

The data collected from the DSC analysis are shown in Table 3. The data are shown in a rising temperature order, with consecutive numerals when the transition is of the same kind. It can be observed that Tg—glass transition temperature, Tm—melting temperature, and Tcc—cold crystallization temperature. Additionally, the crystallization temperature (Tc) and its enthalpy (ΔHc) were evaluated from the cooling run.

The heating of PU allowed us to observe one glass transition with Tg −38.4 °C and one melting point at 54.9 °C [24]. The DCS measurements of P3HB indicated one glass transition with Tg 5.5 °C and two melting points during heating at the temperatures of 157.5 °C and 167.8 °C. The additive of 10% by mass of PU (blend C10) resulted in the presence of two glass transitions; one was shifted below the Tg of P3HB and the second below the Tg of PU. Additionally, the C10 composition showed two melting points, i.e., two-phase systems created the interpenetrating polymer networks. P3HB and PU interact by hydrogen bonds, whose presence was confirmed by FTIR analysis, and it was also discussed in our previous paper [24]. It is the system with the least tendency to crystallize, which is proved both with the crystallization enthalpy from the cooling rate (Table 3) and the most intensive cold crystallization measured by the generated heat of the process.

**Figure 7:**
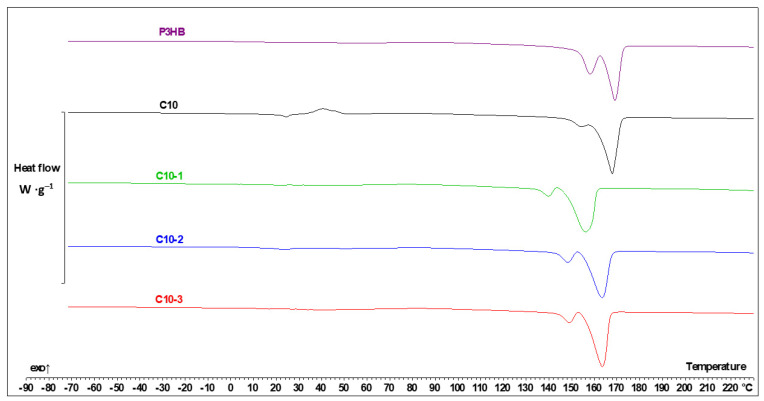
DSC thermal curves of P3HB, P3HB-PU (C10) polymer composition, and polymer nanobiocomposites with 1, 2, and 3 wt.% Cloisite 30B (C10-1, C10-2, and C10-3, respectively) upon heating the samples at 10 °C/min after prior cooling at the same rate.

The addition of the Cloisite 30B in all tested concentrations facilitates the crystallization process (higher heat of this phase transition) and acts like a compatibilizer, which is proven by the disappearance of the glass transition characteristic for the P3HB. For better clarity, the chosen thermal curves are shown in Figure 7. Figure 7 shows the dependence of the experimental heat flow rate of P3HB and the P3HB-PU composition on the temperature obtained based on the heating rate of 10 °C∙min^−1^ at the temperature range from −90 °C to 230 °C, after the previous cooling at the same rate at the temperature range from 230 °C to −90 °C.

The C10-1 composite (Figure 7) is very interesting; the glass transition temperature is placed at −26.2 °C, which is between the Tg of P3HB and the Tg of PU. It means that they are a thermodynamically compatible components of the obtained hybrid polymer nanobiocomposite.

Other thermal effects for the Cloisite-enriched samples can be explained by comparison with the P3HB matrix. It is worth noting that cold crystallization in the C10-1 composition, as well as the other nanocomposites, occurs with a lower intensity and at a higher temperature than in the case of the C10 composition. This is presented in Figure 7.

A higher amount of Cloisite 30B caused the glass transition temperature to be close to that of the neat PU, and the melting enthalpy of PU phase was 4–5 times higher than that in the case of C10-1 (nanocomposite with 1% of Cloisite 30B). The 2 and 3% by mass nano-additive also act less efficiently as a nucleation agent, which is again proved by the crystallization enthalpy from the cooling run (Table 2) and the heat of the cold crystallization (Figure 7). This expectedly leads to the higher heat of fusion in the P3HB melting region. It has to be noted though that Cloisite 30B speeds up the formation of the crystalline phase, but the total heat of the fusion of the P3HB part in the C10 sample is slightly higher than in the C10-1, C10-2, and C10-3 samples. In this case, polymers crystallize faster, but to a slightly lower extent.

The introduction of 1% of the organophilic modifier decreases the glass transition temperature of P3HB. It is connected with the nanofiller distribution in the polymer composition matrix. In the C10-1 sample containing smaller amounts of Cloisite30B, clusters and agglomerates (micrograph in 5c) of nanoparticles are visible as well as their separation from the composite matrix. In the case of samples with 2 and 3% by mass nanofiller (SEM micrographs in Figure 5d,e), a uniform distribution of the nanofiller results in a further decrease in the glass transition temperature and a lower tendency to crystallization and better mechanical properties.

### 3.7. Biodegradability of P3HB and Its Composition and Hybrid Nanocomposites

Based on the respirometric biochemical oxygen demand test performed for 28 days, it was found that the native P3HB has a biodegradability degree of 63.21%. This result is very good and suggests the high susceptibility of this material to biodegradation. The obtained degree of biodegradation also confirms that this material is classified as a biodegradable polymer. The biodegradability of the composition containing 90% by mass of P3HB and 10% by mass of linear polyurethane containing aromatic rings (C10) was also tested. The study showed that the introduction of polyurethane material into the composition reduced the biodegradability to 49.36% (Table 4). This was due to the fact that the aromatic structures derived from MDI, which are very difficult to biodegrade, were introduced into the polymer system. Composites obtained on the basis of P3HB containing a linear polyurethane with aromatic rings and layered nanoaluminosilica were characterized by lower values of the degree of biodegradation. The introduction of 10% by mass polyurethane and 1% by mass of the nanoclay Cloisite 30B resulted in a reduction in this parameter from 63.21% to 44.22%. The content of carbon in the tested samples is similar (Table 4 and Table 5), i.e., other factors have an impact on the rate of biodegradation. This is mainly due to two factors. The first one is related to the introduction into the composite of a polyurethane material obtained on the basis of a linear polyether polyol and aromatic MDI isocyanate. Both structures are very durable, both chemically and biologically. Therefore, it can be assumed that they are not subject to biodegradation processes, which significantly reduces the degree of biodegradation. The second factor influencing the reduction in biodegradation is the use of a nanofiller in the form of organomodified montmorillonite. The presence of an inorganic nanofiller dispersed in the P3HB matrix further inhibited the biodegradability of this material in the process. This relationship was confirmed by subsequent samples in which the Cloisite 30B content increased from 1% by mass up to 3% by mass (every 1% by mass). The increase in the amount of nanofiller gradually reduced the biodegradation from 44.22% to 38.62%, which had the highest share of nanofiller.

### 3.8. In Vitro Cytotoxicity Studies

To assess the biocompatibility of the studied materials, we used two human cell lines: BJ skin fibroblasts and immortalized HaCaT keratinocytes. BJs are normal cells from the skin of neonatal males with a long lifespan in comparison to other normal human fibroblast cell lines. HaCaTs are immortalized cells that have a few advantages over primary keratinocytes. Donor variability, a short culture lifetime, and variations between passages are the negative features of the latter. Therefore, HaCaT keratinocytes was our choice due to their lower variability compared to primary keratinocytes, long-term growth in cultures, and no spontaneous tumorigenic properties [45]. Studies showed that BJ fibroblasts and HaCaT keratinocytes are appropriate and excellent tools for toxicity assessment [46].

**Figure 8:**
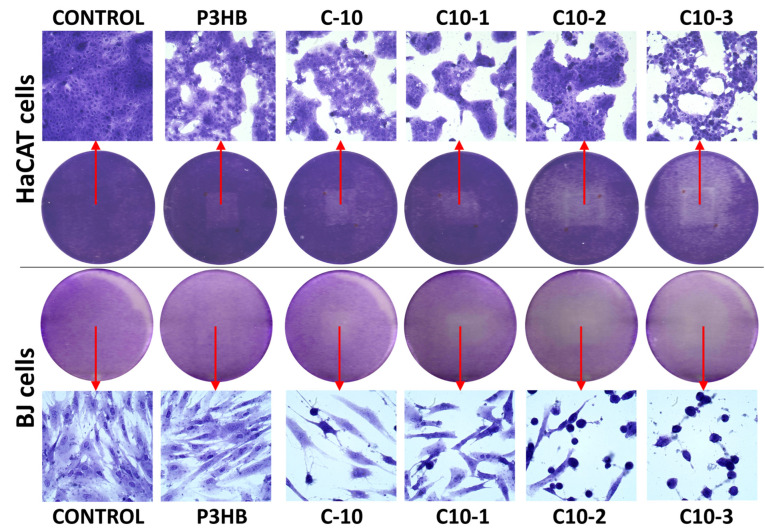
Microscopic images of human fibroblasts (BJs) and immortalized keratinocytes (HaCaTs) stained with crystal violet after 24 h incubation with the studied materials. Round images present the plate wells containing the stained cells, with brighter parts indicating the localization of the samples. The top and bottom rows show cells from the central parts of the reactivity zones.

The tested materials showed a rather low cytotoxicity against the studied cell lines. After 24 h incubation, changes in cell adhesion and shape were observed only in the areas of the P3HB, C10, and C10-1 samples (Figure 8), which allows us to determine that these changes were slight according to the guidelines of the U.S. Pharmacopeial Convention (Table 6). The C10-2 and C10-3 materials demonstrated a higher impact: mild and moderate reactivity was observed, respectively, against both cell lines.

**Figure 9:**
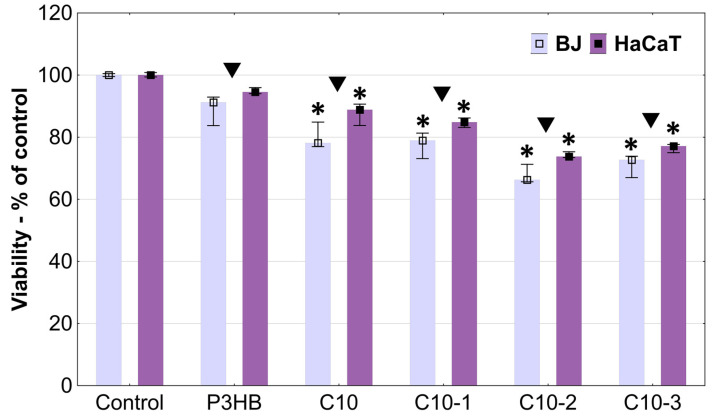
The viability of normal human fibroblasts (BJs) and immortalized human keratinocytes (HaCaTs) after 24 h incubation with the studied samples, estimated with the direct contact assay. Results are expressed as medians. The lower (25%) and upper (75%) quartile ranges are presented as whiskers. Asterisk * indicates differences between the control and samples (*p* < 0.05, Kruskal–Wallis test). Symbol ▼ means significant differences between cell lines for a particular sample (*p* < 0.05, Mann–Whitney U test).

The microscopic observations showed that all tested materials caused a decrease in the confluence of cells located under the samples. A stronger effect was visible in the case of samples C10-2 and C10-3, which additionally caused the shrinkage and loss of adhesion of fibroblasts. Comparing both cell lines, it can be concluded that the fibroblast cells were more sensitive (Figure 8). The absorbance measurements reflected the previously obtained results. The most biocompatible material was the P3HB sample, which did not induce statistically significant changes in the cell viability of both lines compared to the non-treated controls. The first signs of toxicity appeared after the incubation of cells with the C10 sample. The 10% addition of polyurethane turned out to increase toxicity, but to a very small extent. Fibroblast viability decreased only by about 22% and that of HaCaTs by 11% (Figure 9).

P3BH is considered as a biocompatible, non-toxic material that can be used in medicine [47]. Wu et al. indicated that P3HB after extraction and purification with ethanol can be used to made scaffolds by 3D printing. Biocompatibility assessed with HaCaT epidermal cells, confirmed the results of our studies—P3HB was not cytotoxic, did not self-degrade, and allowed a high homogenous cell proliferation of HaCaT cells [48]. It is known that MDI, used in polyurethane synthesis, is not highly biocompatible and its presence may cause skin sensitization [49]. Also, our previous study indicated the toxic action of MDI included in polyurethanes against BJ and HaCaT cells [50]. Therefore, the lowering of the viability of the studied cells was predictable. The addition of Cloisite to the C10 samples caused a further decrease in cell viability, proportional to its concentration. C10-1 decreased BJ and HaCaT cell viability to 79% and 84%, respectively, while C10-2 to 66% and 74%, respectively. The cells incubated with C10-3 had a higher viability compared to C10-2, but only slightly (73% and 77% for BJs and HaCaTs, respectively). The real-time high throughput analyses of exposed cellular systems confirmed that Cloisite 30B induced significant toxic effects, with time-dependent decreases in BEAS-2B human lung epithelial cell viability and alterations in the cell morphology upon exposure [51]. Maisanaba et al. studied the cytotoxicity of Cloisite 30B against a human intestinal Caco-2 cell line after 24 or 48 h exposure with neutral red or tetrazolium salt reduction assays. They showed that Cloisite 30B induced toxic effects at 3.91 µg/mL [52].

Each of the tested materials was significantly more active towards normal fibroblast cells than keratinocytes. Keratinocytes are characterized by a higher resistance due to the fact that they constitute the first barrier separating the organism from environmental factors. A slightly higher sensitivity of fibroblasts compared to keratinocytes, including the immortalized HaCaT line, has also been observed by others [46].

## 4. Conclusions

Hybrid polymer nanobiocomposites were prepared by using natural aliphatic polyester as the matrix, polyurethane with aromatic rings as the modifier, and organomodified montmorillonite as the nano-additive.

The produced hybrid biocomposites had an exfoliated structure, identified by SAXS technique, which leads us to expect better properties of the obtained materials. Nanobiocomposites containing 10% of PU and 2% by mass of Cloisite 30B showed the best mechanical properties, i.e., the highest impact strength (IS) and unchanged hardness compared to the native P3HB and the P3HB-PU polymer composition. The increase in IS attained 80% in comparison with that of the unmodified P3HB. The hybrid nanocomposites were less brittle and had only a slightly smaller tensile strength value, which was still quite high.

A FTIR analysis of the tested materials revealed the characteristic bands of both the polyester matrix and the aromatic polyurethane and confirmed the formation of an interpenetrating polymer network structure, explaining further the increase in the impact strength of the polymeric matrix.

The SEM micrographs of the hybrid nanobiocomposites with 2 wt.% Cloisite 30B and 10 wt.% PU showed a uniform distribution of the nanofiller and the formation of rough domains, arranged in different directions, explaining the better mechanical properties. The SEM micrograph of the native P3HB showed a slightly wavy and glassy surface, typical of brittle polymers, without an elongated structure.

The thermal properties of the P3HB-based hybrid polymer nanobiocomposites with aromatic linear polyurethane and organomodified montmorillonite are comparable to the thermal stability of the P3HB-PU composition.

The presence of PU and Cloisite 30B resulted in a reduction in the biodegradability degree due to their durable structures, both chemically and biologically. Preliminary cytotoxicity studies showed that the P3HB-based hybrid polymer nanobiocomposites with aromatic linear polyurethane and organomodified montmorillonite, which have more favorable physicochemical properties, have a minimally lower biocompatibility than that of P3HB, but still at a satisfactory and acceptable level. Therefore, the obtained materials can be considered for applications connected with the contact with the human body. This issue requires further in-depth studies.

## Figures and Tables

**Table 1:** Composition of the polymer mixtures.

Content (m/m%)			Sample Designation
P3HB	PU	Cloisite^®^	
90	10	0	C10
89	10	1	C10-1
88	10	2	C10-2
87	10	3	C10-3

**Table 2:** Interpretation of the TG and DTG curves of the native P3HB, the one biocomposed with PU, and hybrid nanobiocomposites with PU and Cloisite 30B obtained at a heating rate of 5 °C/min in a nitrogen atmosphere.

Sample	T_on_ (°C)	T_5%_ (°C)	T_10%_ (°C)	T_50%_ (°C)	T_max_ (°C)	Residue at 600 °C (%)
P3HB	221.1	236.2	245.6	281.2	295.7	1.70
C10	252.1	268.8	295.1	295.6	294.5	2.82
C10-1	252.2	272.8	292.5	293.3	293.7	1.38
C10-2	254.8	273.5	295.2	284.5	295.2	1.97
C10-3	252.3	272.3	294.8	283.8	293.3	2.59

T_on_—temperature of the onset decomposition. T_x%_—temperature of the 5wt% of the mass lost. T_max_—temperature of the fastest decomposition.

**Table 3:** Thermal characteristics of P3HB, PU, P3HB-PU (C10) polymer composition, and polymer nanobiocomposites with 1, 2, and 3 wt.% Cloisite 30B (C10-1, C10-2, and C10-3, respectively) upon heating the samples at 10 °C/min after prior cooling at the same rate.

Sample	T_g1_, (°C)	ΔC_p_ (J·g^−1^·°C^−1^)	T_g2_, (°C)	ΔC_p_ (J·g^−1^·°C^−1^)	T_m1_, (°C)	ΔH_f1_ (J·g^−1^)	T_cc_, (°C)	ΔH_cc_ (J·g^−1^)	T_m2_, (°C)	T_m3_, (°C)	ΔH_f_ (J·g^−1^)	T_c_, (°C)	ΔH_c_, (J·g^−1^)
P3HB	-----	-----	5.5	0.148	-----	-----	89.9	-4.76	157.5	167.8	97.4	85.7	−78.6
PU	−38.4	0.145	-----	-----	54.9	111.3	-----	-----	------	-----	-----	23.9	−124.5
C10	−42.9	0.045	−2.9	0.086	24.2	1.35	40.5	−24.4	153.8	166.7	82.1	63.5	−43.8
C10-1	−26.2	0.079	-----	-----	21.9	0.37	79.9	−8.22	139.3	155.3	79.7	59.8	−59.2
C10-2	−45.0	0.050	-----	-----	23.5	2.18	84.8	−9.62	147.7	162.0	75.2	66.4	−56.7
C10-3	−43.8	0.039	-----	-----	44.4	1.47	85.7	−12.1	148.5	162.3	77.5	70.2	−58.1

**Table 4:** Measured values of the biochemical oxygen demand of the tested samples and determined values of the theoretical oxygen demand and the degree of biodegradation.

Sample	Sample Mass (g)	TOD (mg/L)	Determined BOD (mg/L)	BOD of Sample (mg/L)	D_t_—Degree of Biodegradability (%)
P3HB	0.25	49.84	60.00	31.50	63.21
C10	0.15	84.08	70.00	41.50	49.36
C10-1	0.25	50.20	50.70	22.20	44.22
C10-2	0.23	54.31	50.60	22.10	40.70
C10-3	0.24	51.79	48.50	20.00	38.62

**Table 5:** The share of individual elements in the composites calculated on the basis of the chemical structure of the components and determined on the basis of elemental analysis.

Sample	C (%)		H (%)		O (%) Calculated	Si (%) Calculated	N (%)	
	Calculated	Determined	Calculated	Determined			Calculated	Determined
P3HB	55.81	55.95	6.98	6.93	37.24	0.00	0.00	0.79
C10	55.65	55.62	7.15	7.07	36.40	0.00	0.80	0.82
C10-1	55.60	55.40	6.99	7.04	36.55	0.47	0.42	0.83
C10-2	55.04	55.04	6.92	7.02	36.71	0.93	0.42	0.80
C10-3	54.48	54.73	6.85	6.99	36.87	1.40	0.42	0.76

**Table 6:** Reactivity grades for the direct contact test according to the U.S. Pharmacopeial Convention [33].

Grade	Reactivity	Description of Reactivity Zone
0	None	No detectable zone around or under specimen
1	Slight	Some malformed or degenerated cells under specimen
2	Mild	Zone limited to area under specimen and less than 0.45 cm beyond specimen
3	Moderate	Zone extends 0.45–1.0 cm beyond specimen
4	Severe	Zone extends more than 1.0 cm beyond specimen

## Data Availability

The original contributions presented in the study are included in the article, further inquiries can be directed to the corresponding authors.
